# Machine Learning-Guided Design of MQ Silicone Resin Reinforced Addition-Curing Silicone Rubber: From Literature Data Mining to Experimental Validation

**DOI:** 10.3390/polym18182204

**Published:** 2026-09-10

**Authors:** Tianyi Xu, Yuewen Huang, Hui Liu, Dan Qiu, Yuan Yuan, Shuaitao Zhang, Bin Wang

**Affiliations:** 1 Guangzhou Institute of Chemistry, Chinese Academy of Sciences, Guangzhou 510650, China; xutianyi23@mails.ucas.ac.cn (T.X.);; 2 University of Chinese Academy of Sciences, Beijing 100049, China; 3 CAS Engineering Laboratory for Special Fine Chemicals, Guangzhou 510650, China; 4 CASH GCC Fine Chemicals Incubator (Nanxiong) Co., Ltd., Nanxiong 512400, China; 5 CASH GCC Shaoguan Research Institute of Advanced Materials, Shaoguan 512400, China

**Keywords:** MQ silicone resin, addition-curing liquid silicone rubber, machine learning, random forest, literature data mining, mechanical property prediction, experimental validation

## Abstract

MQ silicone resins are widely used reinforcing fillers for addition-curing liquid silicone rubber (LSR); however, establishing a quantitative composition–property relationship remains challenging because published data are fragmented across matrix chemistries, crosslinkers, catalyst systems and testing standards. Here we present a machine-learning-guided workflow integrating literature data mining, interpretable random-forest (RF) modelling and independent experimental validation for the design of MQ-reinforced LSR. An RF model trained on 55 curated literature points spanning RTV and LSR systems, using four physically motivated descriptors (MQ content, M/Q ratio, curing system and vinyl content), yielded leave-one-out coefficient of determination (R^2^) values of 0.741 for tensile strength (TS) and 0.730 for Shore A hardness (HA), with mean absolute errors of 0.63 MPa and 8.95 ShA, respectively. Feature-importance and partial-dependence analyses identified MQ content as the dominant descriptor. Guided by the model, seven LSR formulations (vinyl content 4 wt%, M/Q = 0.8, loading 5–35 wt%) were designed and fully characterised: the model reproduced the measured TS and HA for all seven formulations within the corresponding training mean-absolute-error tolerance, whereas elongation at break (EB), whose prediction is substantially weaker (LOO R^2^ ≈ 0), was captured only as a qualitative trend with respect to MQ loading. This workflow demonstrates that a modest, curated literature dataset, mined by an interpretable ML model, can support formulation design and independent experimental validation—an efficient, low-cost alternative to trial-and-error optimisation.

## 1. Introduction

Silicone rubber (SR) occupies a unique position among elastomeric materials because of its exceptionally broad performance envelope: outstanding thermal stability over a service range of approximately −60 to 250 °C, retention of low-temperature flexibility, high dielectric strength and volume resistivity, low surface energy, superior chemical and weathering inertness, and well-documented biocompatibility. These attributes make it indispensable across a remarkably wide span of engineering sectors, from aerospace sealants, automotive gaskets and high-voltage cable accessories to LED and power-module encapsulation, medical implants, baby products, cookware and protective coatings. Nevertheless, the low intrinsic mechanical strength of the uncrosslinked poly(dimethylsiloxane) (PDMS) backbone (typically below 0.3 MPa in tensile strength and far too soft for structural duty) renders silicone rubber unsuitable for most load-bearing applications without reinforcement. This inherent weakness traces back to fundamental molecular features: the weak intermolecular van der Waals interactions between the methyl-rich chains, the high conformational flexibility and the very low rotational barrier of the Si–O–Si backbone, and the consequently low cohesive energy density and weak chain entanglements, which together prevent the formation of a mechanically robust three-dimensional network even after chemical crosslinking. Reinforcement is therefore not merely an incremental improvement but an indispensable prerequisite for the practical deployment of silicone materials in demanding structural and electronic applications [1,2,3].

To overcome this intrinsic limitation, a variety of reinforcing strategies have been developed, each offering a distinct balance between reinforcement efficiency, processability, and cost. Nanoscale fumed and precipitated silica remain the most widely adopted fillers; their reinforcement derives from strong hydrogen bonding between surface silanol groups and the PDMS backbone and from the construction of a percolated filler network that partitions stress across the matrix [4,5,6]. However, silica is notoriously difficult to disperse in the hydrophobic PDMS medium, frequently requiring extensive surface hydrophobisation, mechanical shearing, and even azeotropic water removal, and its incomplete dispersion leads to phase separation, increased viscosity, and residual hydrophilicity that can degrade long-term electrical performance. Carbonaceous nanofillers such as carbon nanotubes and graphene have been explored as hybrid reinforcement agents that simultaneously confer electrical conductivity, yet their high cost, poor dispersion, and pronounced mechanical anisotropy restrict them to niche applications. Against this backdrop, MQ silicone resins have emerged as a highly attractive alternative: being siloxane-based and therefore chemically homologous to the PDMS matrix, they are intrinsically compatible, easy to incorporate, and capable of delivering pronounced reinforcement without the dispersion and dielectric penalties that plague conventional particulate fillers [7,8].

MQ silicone resins are a distinct class of highly branched oligomeric siloxanes whose nomenclature encodes the functionality of the constituent building blocks: M denotes monofunctional trimethylsiloxy (R3SiO1/2) units that cap and solubilise the structure, while Q denotes tetrafunctional tetrasiloxy (SiO_4_) cores that impart rigidity and connectivity, with the molar M/Q ratio dictating the overall molecular architecture and the size, density and compactness of the Q domains [7,8,9,10]. From a mechanical standpoint the resin behaves as a rigid, covalently crosslinked nanodomain: the Q-rich core provides load-bearing rigidity and stress transfer, whereas the flexible, methyl-dense M shell confers molecular-level compatibility with the PDMS matrix, eliminating the dispersion and interfacial problems associated with inorganic fillers. Because the matrix and the filler share an identical siloxane chemistry, MQ-reinforced systems avoid the sharp matrix–filler interfacial energy mismatch that complicates silica-filled rubbers, and the resulting composites retain optical transparency even at moderate resin loadings, a property of particular value for LED encapsulation and optical-grade components. The M/Q ratio is thus the primary structural lever: too low an M/Q yields dense, brittle, poorly dispersing cores, whereas too high a ratio produces soft, loosely crosslinked domains with limited reinforcing efficiency [7,9,10].

When vinyl-containing M units are incorporated, the pendant vinyl groups participate in platinum-catalysed hydrosilylation during addition curing, covalently tethering the resin to the surrounding PDMS network and thereby converting a passive filler into an integrated reinforcing scaffold that is fused into the elastomer network rather than merely embedded within it [11]. This covalent integration allows stress to be transferred directly from the deformable matrix into the rigid Q domains through chemical bonds rather than weak physical contacts, which can elevate the tensile strength of addition-curing LSR to 6–8 MPa under optimised conditions, a level that purely physical blending cannot attain [12,13,14]. The net reinforcement is governed by three coupled structural descriptors: the M/Q molar ratio, which controls the size, rigidity and primary particle strength of the Q domain; the vinyl content, which fixes the density of covalent anchoring points between the resin and the matrix and thus the crosslink density of the composite; and the resin concentration, which sets the filler volume fraction and the interparticle spacing. These descriptors interact in an inherently nonlinear fashion: an elevated M/Q ratio softens the resin core and curtails reinforcement, an excessive vinyl content over-crosslinks the network and embrittles the composite at the expense of elongation, and an overly high resin loading can lead to aggregation and network imperfection. Their joint, simultaneous optimisation therefore lies at the heart of rational formulation design, yet it is precisely this multiparameter coupling that makes empirical optimisation so difficult [13,14].

Despite more than two decades of intensive experimental work, translating the accumulated body of knowledge into rational formulation design remains remarkably difficult because the published data are highly fragmented and heterogeneous. Each study employs a different matrix polymer, ranging from high-viscosity, condensation-curing, two-part RTV pastes to low-viscosity, addition-curing LSR systems; MQ resins that are prepared by different synthetic routes, terminated with different functional groups and stored under different conditions; different crosslinkers, catalyst types and catalyst loadings; and different curing temperatures, times and mechanical-testing standards. As a consequence, identical experimental conditions are rarely reproduced across laboratories, absolute property values are frequently presented only as figures without tabulated numerical data, and reported elongations and hardnesses are sensitive to sample geometry, crosshead speed and ageing history. Manually comparing or aggregating such scattered results is error-prone, and a consistent quantitative structure–property relationship cannot be extracted by inspection alone. Conventional trial-and-error optimisation is simultaneously time-consuming and resource-intensive, typically requiring dozens of iterative synthesis–characterisation cycles per composition and consuming significant quantities of expensive platinum catalyst and specialty reagents. This fragmentation and the non-transferability of isolated conclusions strongly motivate a data-centric strategy in which the scattered published results are consolidated, cleaned, standardised and mined jointly to expose trends that remain invisible in any single study [15,16,17,18,19].

Machine learning (ML) has repeatedly proven effective at extracting robust predictive relationships from noisy, multi-source datasets across materials science and polymer informatics [20]. In domains ranging from the prediction of glass-transition temperatures, dielectric constants, and mechanical properties of polymers [21,22,23,24] to the screening of catalyst and formulation spaces and the accelerated discovery of organic functional materials, data-driven models have consistently outperformed purely empirical heuristics [25,26,27] once a sufficiently standardised corpus of data is assembled. Among the available algorithms, the random forest (RF) is especially well suited to small, heterogeneous datasets because of its robustness against overfitting, its natural handling of mixed continuous and categorical descriptors without scaling, and its interpretability through built-in feature-importance and partial-dependence diagnostics [28]. While deep neural networks demand large sample sizes and meticulous hyperparameter tuning and behave as black boxes, tree-ensemble models such as RF deliver stable, physically interpretable predictions even when the training set contains only a few dozen points—precisely the small-data regime encountered in siloxane materials informatics, where synthesising a single composition is far more costly than generating a data point, and for which dedicated strategies have been developed [22]. A targeted literature search confirms that, although ML has been applied broadly in polymer science, its specific application to the industrially important question of MQ-resin-reinforced silicone rubber remains essentially unexplored, leaving a clear and valuable niche for the present work [25,26,27].

**Figure 1:**
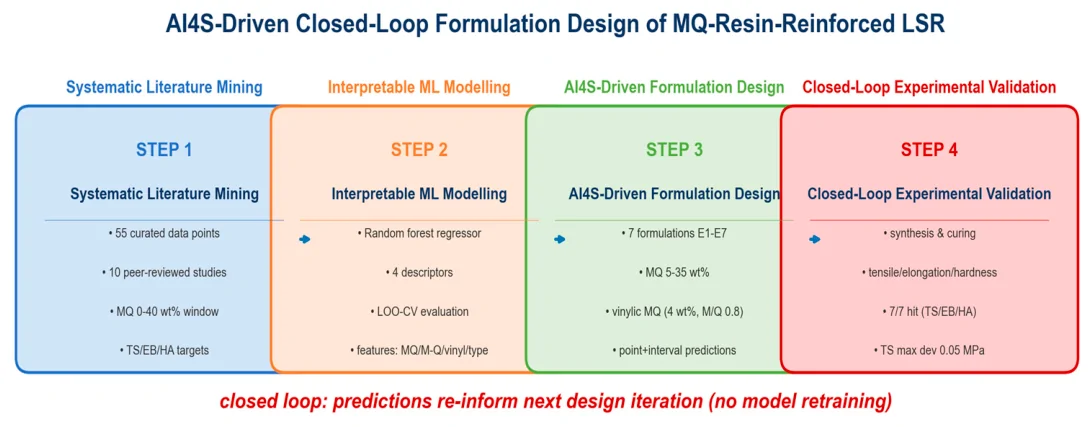
Schematic of the machine-learning-guided workflow comprising literature mining, ML modelling, model-driven formulation design and independent experimental validation.

In this study, we present a machine-learning-guided workflow, summarised schematically in Figure 1. First, a systematic literature mining and curation step assembles 55 mechanical-property data points on MQ-reinforced silicone rubber, drawn from ten distinct published studies that together span both condensation-curing RTV and addition-curing LSR chemistries and a wide window of MQ loadings, M/Q ratios and vinyl contents. Second, an interpretable random-forest model is trained on this curated dataset to predict tensile strength (TS), elongation at break (EB) and Shore A hardness (HA) as functions of MQ content, M/Q ratio, curing-system type and vinyl content, and its predictive limits and governing descriptors are quantified rigorously through leave-one-out cross-validation and through a comparison against simpler linear baselines. Third, guided by the model predictions, seven new addition-curing LSR formulations containing a vinyl-functionalised MQ resin (vinyl content 4 wt%, M/Q = 0.8) are designed across a wide MQ loading range of 5–35 wt%. Finally, all seven formulations are synthesised, cured and fully characterised, and the measured properties are compared quantitatively with the model predictions. The model quantitatively reproduced the measured TS and HA for all seven formulations within the corresponding training mean-absolute-error tolerance, whereas EB was captured only as a qualitative trend. This workflow (literature mining, ML prediction, rational design and independent experimental validation) provides a practical and quantitative route to vinyl-functionalised MQ-reinforced silicone material design, and constitutes an efficient, low-cost alternative to conventional trial-and-error formulation optimisation.

## 2. Materials and Methods

### 2.1. Literature Data Mining and Curation

A systematic literature search was conducted to identify peer-reviewed studies reporting quantitative mechanical properties of MQ-resin-reinforced silicone rubber, with particular emphasis on addition-curing LSR formulations containing vinyl-functionalised MQ resins. The search covered a representative cross-section of the field, including studies of MQ-resin self-reinforced room-temperature-vulcanised (RTV) silicone rubber, vinyl-MQ-resin-reinforced liquid silicone rubber (LSR), MQ-resin-reinforced RTV liquid silicone rubber, the influence of MQ resin preparation conditions on RTV reinforcement, high-strength RTV systems prepared from MQ resins, and addition-cured PDMS composites filled with nano-silica sol together with MQ resin. Together these ten contributing studies, spanning both the Chinese-language and international literature, provide the composition–property coverage necessary to span a meaningful fraction of the formulation space rather than a single isolated composition–property pair.

Because multi-source data are inherently inconsistent in reporting style, a rigorous quality screen was applied before any data point entered the training set. Three exclusion rules were enforced. First, each retained entry was cross-checked for internal consistency between the stated composition and the reported mechanical values, and entries whose tabulated value disagreed with the narrative text, or which were clearly mis-transcribed in the original, were discarded. Second, duplicate measurements that appeared in more than one series or in more than one publication were retained only once to avoid biasing the model towards a single laboratory. Third, entries based on methyl-terminated (non-vinyl) MQ resins were excluded, because such resins reinforce through a fundamentally different mechanism (physical entanglement without covalent anchoring) and would otherwise introduce an uncontrolled systematic offset. Application of these criteria reduced the raw harvest to 55 mutually consistent, physically comparable data points. The curing system (addition vs. condensation) was encoded explicitly as a categorical descriptor, rather than being allowed to act as a confounding variable.

The resulting dataset spans a wide and practically relevant window of formulation space: MQ contents from 0 to 50 wt%, M/Q molar ratios from 0.75 to 1.3, vinyl contents up to 7.6 wt%, and both condensation-curing RTV and addition-curing LSR chemistries. As summarised in Table 1, the recorded tensile strengths range from 0.18 to 7.2 MPa (mean 1.78 MPa), elongations at break from 23.9% to 1001% (mean 161%) and Shore A hardnesses from 12 to 84 ShA (mean 40 ShA); the smaller sample sizes for elongation (*n* = 51) and hardness (*n* = 39) reflect the omission of entries for which these properties were not reported. This limited data volume is an inherent feature of domain-specific literature mining rather than a weakness of the curation protocol: because each study employs a different matrix polymer, MQ resin synthetic history, crosslinker chemistry, and testing protocol, only a minority of published measurements are directly comparable. Small-data regimes are nevertheless a recognised and well-studied feature of materials informatics [22] and each retained point carries correspondingly higher information content than an equivalent entry in a large generic database.

**Figure 2:**
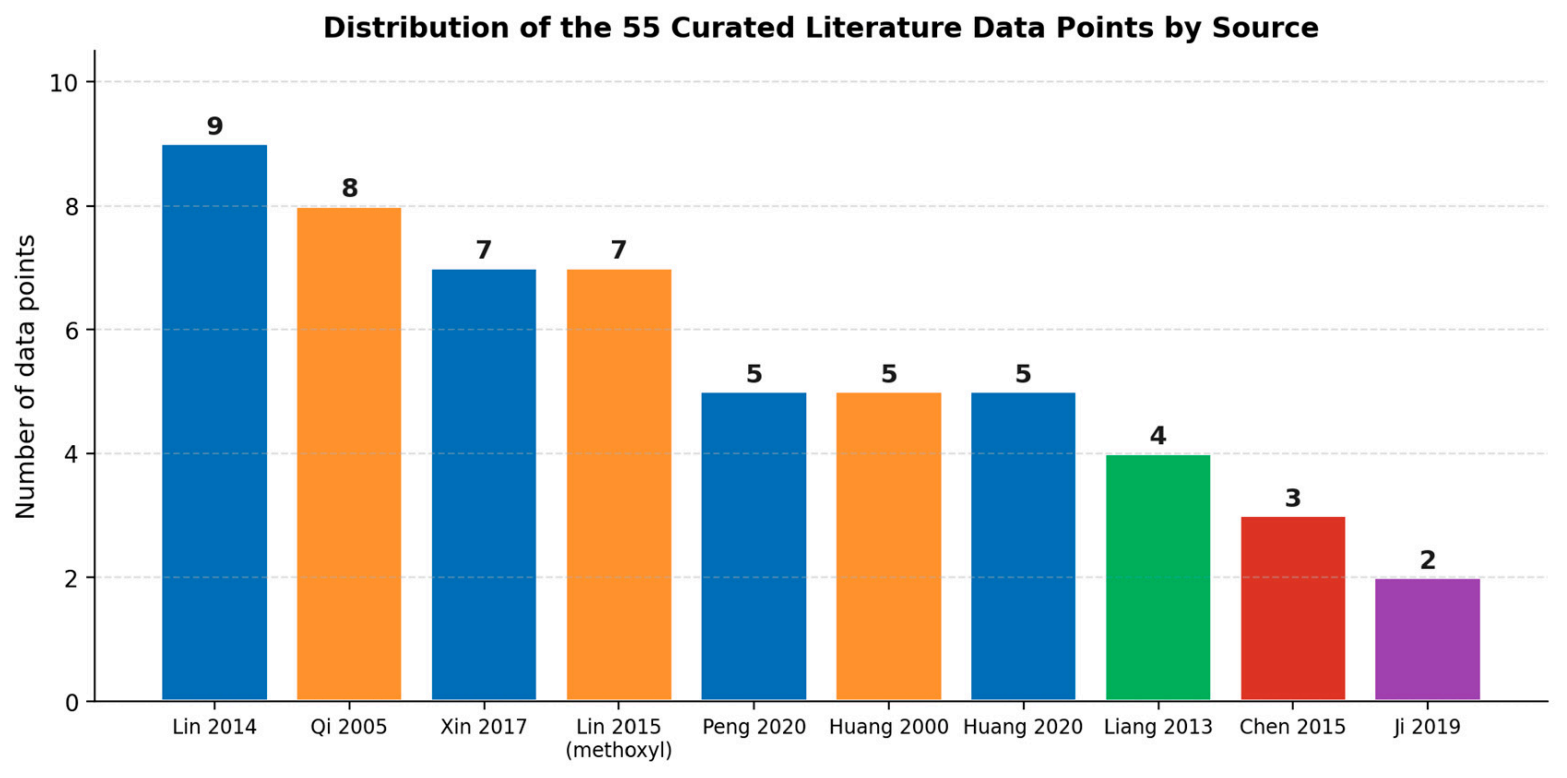
Distribution of the 55 curated literature data points among the contributing studies [15,16,17,18,19].

The composition of the curated dataset across the ten contributing studies is visualised in Figure 2; the individual studies contribute between two and nine data points each, ensuring that no single source dominates the model and that the learned structure–property relationship reflects an aggregate of independent experimental evidence rather than the bias of any one laboratory.

### 2.2. Random-Forest Model

A random-forest (RF) regressor was implemented using the scikit-learn library. RF is an ensemble method that builds a large collection of decision trees, each fitted on a bootstrap resample of the training data and on a random subset of the descriptors, and then averages their individual predictions. This bagging plus feature-subsampling strategy decorrelates the individual trees, suppresses the high variance that plagues a single deep decision tree, and yields a stable, low-bias estimator that is robust to overfitting, an essential property when the training set comprises only a few dozen points. To guarantee full reproducibility, the ensemble was configured with n_estimators = 500 and a fixed random state of 42, retaining the scikit-learn defaults for the remaining hyperparameters; deliberately restricted to only n_estimators and random_state, the configuration is transparent and exactly matches the training script released as Supplementary Material. The deliberate choice of a compact, physically motivated feature set of four descriptors, namely MQ content, M/Q molar ratio, curing-system type, and vinyl content, reflects the three structural levers identified in the Introduction as governing reinforcement, together with the matrix chemistry; this restricted input space avoids overfitting in the small-data regime and preserves the physical interpretability of the feature-importance ranking obtained from the trained model (Section 3.2). The linear models used as baselines (Section 3.1) were the ordinary least-squares multiple linear regression (MLR) and ridge regression; a Gaussian process regressor (GPR) with a radial-basis kernel was also evaluated to probe the small-sample behaviour of a kernel method.

Generalisation performance was assessed by leave-one-out cross-validation (LOO-CV), in which the model is trained on n − 1 samples and evaluated on the single withheld sample, the procedure being repeated until every sample has served once as the validation point. For a dataset of only 55 entries, LOO-CV is preferable to a fixed train/test split or to a standard k-fold procedure, because it maximises the amount of data available for training at each step and thereby provides a low-variance, close-to-unbiased estimate of how the model will perform on genuinely unseen formulations. Three complementary regression metrics were computed: the coefficient of determination (R^2^), which quantifies the fraction of total variance explained; the mean absolute error (MAE), which measures the average magnitude of the prediction error in the original physical units and is robust to outliers; and the root mean squared error (RMSE), which additionally penalises large deviations. Together these metrics characterise both the overall fit quality and the expected prediction tolerance of the model, the latter being used directly to define the ±MAE tolerance band within which a measured value is judged to reproduce the model prediction (Section 3.3).

The adopted hyperparameter configuration, together with its physical and statistical rationale, is summarised in Table 2. The number of trees was set to 500 to ensure that the ensemble prediction had fully converged, while the remaining hyperparameters were retained at their scikit-learn defaults, with overfitting guarded against by leave-one-out cross-validation and by the deliberately compact four-descriptor feature set. The fixed random state guarantees that every result reported in this work is exactly reproducible by an independent reader.

### 2.3. Materials and Formulation Design

The addition-curing LSR system investigated here comprises four functional components: a vinyl-terminated poly(dimethylsiloxane) (Vi-PDMS) base polymer carrying 0.14 wt% vinyl groups (Shanghai Macklin Biochemical Co., Ltd., Shanghai, China); a vinyl-functionalised MQ resin with a vinyl content of 4 wt% and an M/Q molar ratio of 0.8, which serves as the reinforcing agent (Shandong Dayi Chemical Co., Ltd., Yantai, China); a hydrogen-functionalised PDMS crosslinker with a silicon-hydride content of 0.34 wt% (Shanghai Aladdin Biochemical Technology Co., Ltd., Shanghai, China); and a Speier catalyst (chloroplatinic acid in isopropanol; Guangzhou Institute of Chemistry Co., Ltd., Chinese Academy of Sciences, Guangzhou, China) loaded at a modest 15 ppm of platinum. This particular material platform was chosen deliberately so that every experimental composition falls well inside, rather than beyond, the descriptor ranges spanned by the literature training set: vinyl contents near 4 wt%, M/Q ratios around 0.8, and MQ loadings up to 35 wt% are all well represented among the 55 curated points, so the model is interpolating within its validated domain rather than extrapolating into an uncharacterised region. Hydrosilylation chemistry is driven at a Si-H:Si-Vi molar ratio of 1.2, an excess of hydride that ensures complete consumption of the vinyl groups and reproducible network stoichiometry across all compositions. Toluene (Merck, via Merck China, Shanghai, China) was used as a processing aid to dissolve the resin and homogenise the blend before crosslinking; its amount was graded with the MQ loading (3–10 mL per 50 g batch) to maintain a constant effective solid content across the series.

The seven formulations E1–E7 were generated directly and exclusively from the model predictions, without further empirical adjustment and without iterative retraining, in order to test the methodology honestly. The MQ loading was scanned across 5, 10, 15, 20, 25, 30 and 35 wt%, a range that brackets the reinforcement threshold observed in the partial-dependence analysis while staying well within the validated training window. At each loading, the model supplied a point prediction together with a ±MAE tolerance band for TS, EB and HA; the complete set of compositions, including the individual masses of Vi-PDMS, MQ resin, crosslinker and toluene, is listed in Table 3. The choice of a monotonic 5–35 wt% sweep was intentional: it allows the model’s predicted strengthening trend to be checked against the experiment at every intermediate loading, converting a set of isolated point predictions into a stringent, continuously constrained test of the entire structure–property relationship rather than a handful of independent lookups.

The compositions summarised in Table 3 reveal how a constant effective solid content is maintained through the graded toluene dosage, so that any drift in measured properties across the series reflects genuine differences in MQ reinforcement rather than changes in viscosity or filler packing. All batches were formulated to a fixed total mass of 50 g, which fixes the absolute reagent masses and thereby simplifies both weighing and cross-checking during preparation.

### 2.4. Synthesis, Curing and Characterisation

In a typical preparation, the MQ resin was first dissolved in the prescribed volume of toluene under gentle stirring to obtain a clear, homogeneous resin solution; the vinyl-terminated PDMS base polymer was then added, followed by the hydrogen-functionalised crosslinker, and the mixture was stirred until visually homogeneous. Because the hydride-bearing crosslinker can participate in premature crosslinking once the catalyst is present, the Speier catalyst solution was added last, immediately before casting, in order to maximise the open working time. The fully formulated and degassed mixture was cast into moulds and cured at 125 °C for 90 min, conditions that were kept identical for all seven formulations so that property differences arise solely from composition, not from processing history. The curing protocol was verified to be complete by tactile and dimensional stability, with no residual tackiness detected at the end of the cycle.

**Figure 3:**
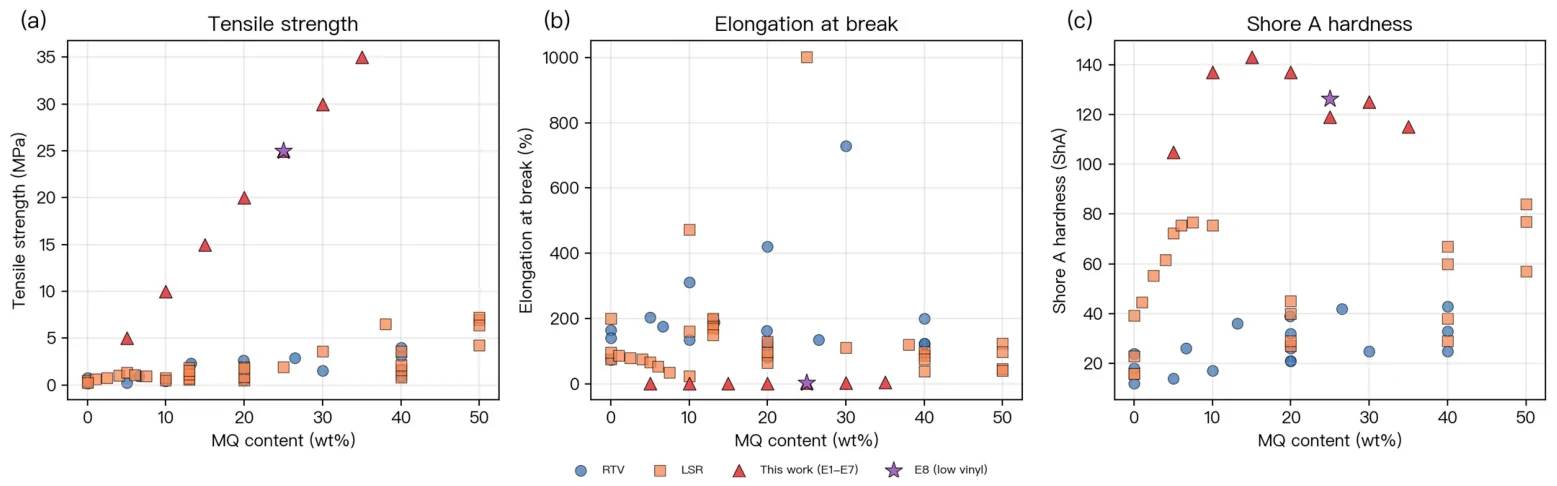
Distribution of literature training data (diamonds) and E1–E7, E8 experimental results (triangles) for (**a**) tensile strength (MPa), (**b**) elongation at break (%), and (**c**) Shore A hardness (ShA).

Mechanical characterisation followed standard protocols. Tensile strength and elongation at break were measured on dumbbell specimens using a universal testing machine (Dongguan Lice Electronics Technology Co., Ltd., Dongguan, China) at a fixed crosshead speed, and Shore A hardness was determined with a conventional durometer (Nanjing Jueyu Precision Instrument Co., Ltd., Nanjing, China) on stacked test plaques. Five specimens (*n* = 5) were tested per formulation, and the results are reported as the mean ± standard deviation (SD). These three properties (TS, EB and HA) were chosen because they are the same quantities predicted by the model, which makes the experimental-comparison step in Section 3.3 a direct, quantitative test of the predictive framework, and because they capture the two competing aspects of reinforcement: the gain in load-bearing capacity (TS, and to a lesser extent, HA) and the retention of deformability (EB); the position of these (later-measured) E1–E8 values relative to the literature training data on each of the three property axes is shown in Figure 3.

## 3. Results and Discussion

### 3.1. Random-Forest Model Performance

**Figure 4:**
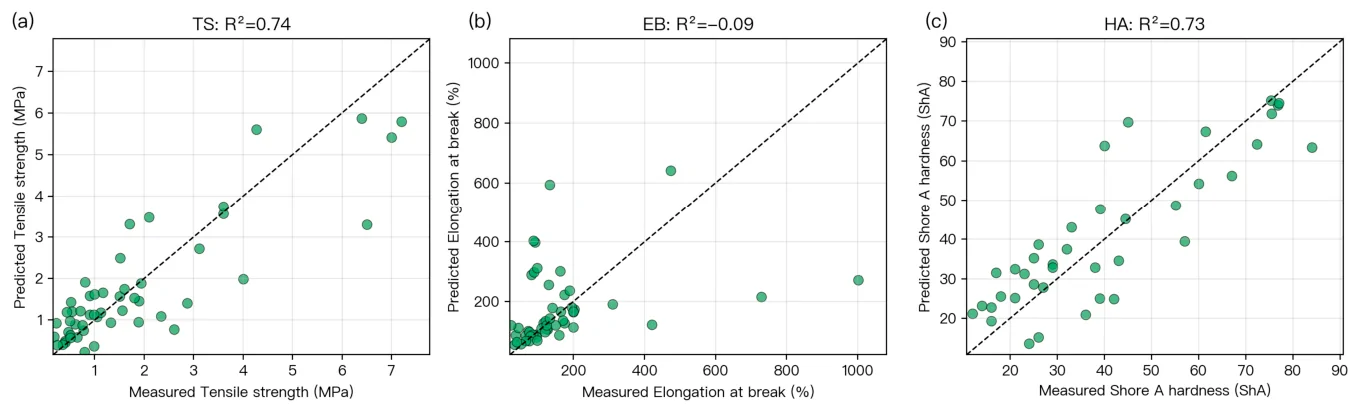
Leave-one-out cross-validation parity plots for (**a**) tensile strength (MPa), (**b**) elongation at break (%), and (**c**) Shore A hardness (ShA). Dashed lines represent perfect prediction.

The LOO-CV results (Table 4) reveal a clear and physically meaningful hierarchy in predictive accuracy. For tensile strength the model attains R^2^ = 0.741 with an MAE of 0.63 MPa and an RMSE of 0.90 MPa; for Shore A hardness it attains R^2^ = 0.730 with an MAE of 8.95 ShA and an RMSE of 10.74 ShA; for elongation at break, however, the coefficient of determination is not significantly different from zero (R^2^ ≈ −0.09), indicating that the model captures essentially none of the variance in EB across the heterogeneous literature corpus. The parity plot in Figure 4 for TS shows most points clustered close to the diagonal, with the scatter increasing towards the high-strength tail, exactly as expected for a small training set in which the extreme end of the property range is sparsely populated. The contrast between the accurate TS and HA predictions and the unsatisfactory EB prediction is instructive rather than accidental: tensile strength and hardness are dominated by the collective network density and the volumetric presence of the rigid MQ phase, both of which are well encoded by the MQ-loading descriptor; elongation, by contrast, depends sensitively on the distribution of crosslinks, the entanglement structure and, in particular, on the exact curing chemistry and vinyl/crosslinker stoichiometry, which are only coarsely represented in the four descriptors. The model is therefore best interpreted as a reliable quantitative guide for strength- and stiffness-dominated design targets, whereas EB should be treated only as a qualitative trend in the small-data regime, a limitation that is examined critically in Section 5.

To place these LOO results in context and to justify the choice of a nonlinear model, the random-forest regressor was benchmarked against three baselines trained on the same curated dataset and evaluated by the same leave-one-out protocol: multiple linear regression (MLR), ridge regression and Gaussian process regression (GPR). The comparison is summarised in Table 5. For tensile strength, RF (R^2^ = 0.741) clearly outperforms the two linear baselines (MLR 0.591, Ridge 0.581) and GPR (0.153), demonstrating that the composition–property relationship is distinctly nonlinear and cannot be adequately captured by a linear model. The same ordering holds for Shore A hardness (RF 0.730 vs. MLR 0.451, Ridge 0.445, GPR 0.176). Crucially, no model—linear or nonlinear—can predict elongation at break quantitatively (all R^2^ ≈ 0), confirming that the weak EB prediction reflects inherent scatter in the heterogeneous literature corpus rather than a deficiency in the chosen algorithm. GPR, which typically requires larger datasets and careful kernel selection, performs markedly worse on the 55-point set, supporting the robustness of a tree-ensemble approach for this small-sample regime.

**Figure 5:**
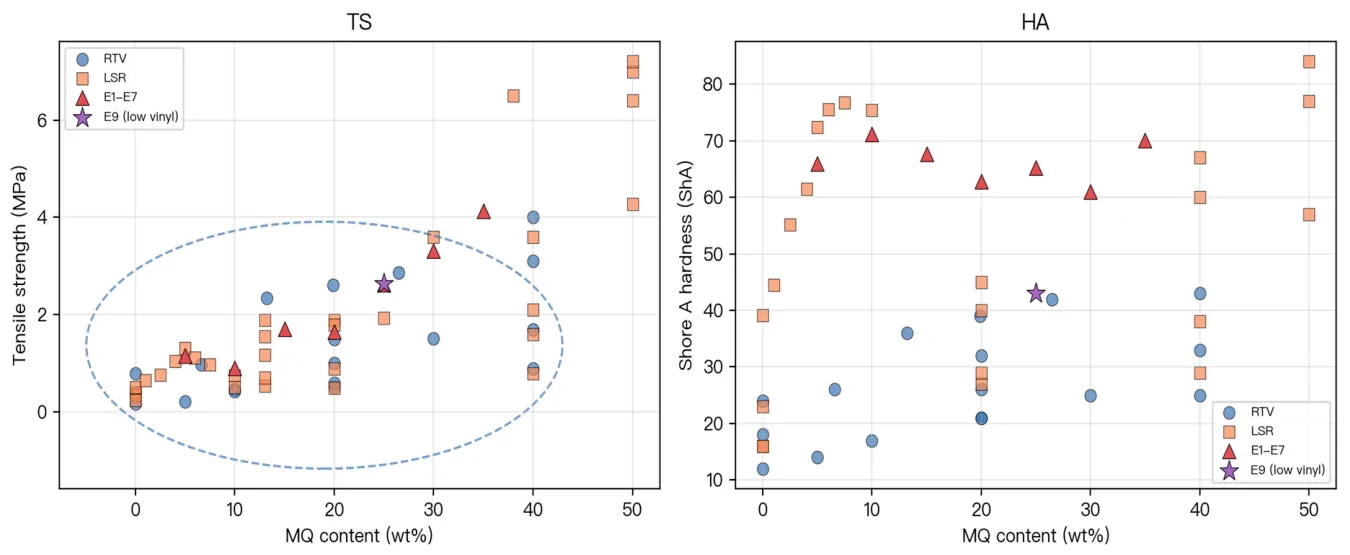
Comparison of MQ-resin-reinforced condensation-curing RTV and addition-curing LSR: literature data (blue circles) and E1–E7, E8 experimental results (red triangles). Dashed ellipses highlight the two system families.

Within this literature-to-experiment framework, trends extracted across the corpus remain physically robust even where absolute comparability is imperfect. Figure 5 compares the two families of experimental systems: condensation-curing RTV rubbers and addition-curing LSR composites. The LSR family, in which vinyl-MQ resin participates covalently in hydrosilylation, systematically reaches higher tensile strengths at a comparable MQ loading than the RTV family, whose reinforcement relies on physical dispersion of a hydroxyl-terminated resin. The clear separation between the two clouds, reproduced by the model through its categorical system-type descriptor, confirms that the chemistry of matrix–resin bonding (covalent anchoring versus physical blending) is a first-order determinant of reinforcement efficiency, and vindicates the decision to treat curing-system type as a model input rather than to average it away.

### 3.2. Model-Driven Formulation Design

The trained RF model was used to guide the design of the validation formulations. For each candidate MQ loading the model returned simultaneous predictions of TS, EB and HA together with the training mean-absolute-error (MAE) tolerance band established in Section 2.2. Instead of proposing a single optimum, which would be premature given the limited data, the model was queried across the full 5–35 wt% window so that the predicted strengthening trajectory could be visualised and then tested point-by-point experimentally. This strategy uses the machine-learning model as a design tool whose predictions are then verified against genuinely new materials: each experimental point E1–E7, E8 simultaneously validates the model on previously unseen compositions. Critically, no descriptor outside the four model inputs was adjusted during this process, and the model was not retrained or iterated after the experiments; the design and the validation were therefore independent. The resulting seven compositions, with full reagent masses, are given in Table 3, and the model-driven composition E8 is described in Section 3.3.

**Figure 6:**
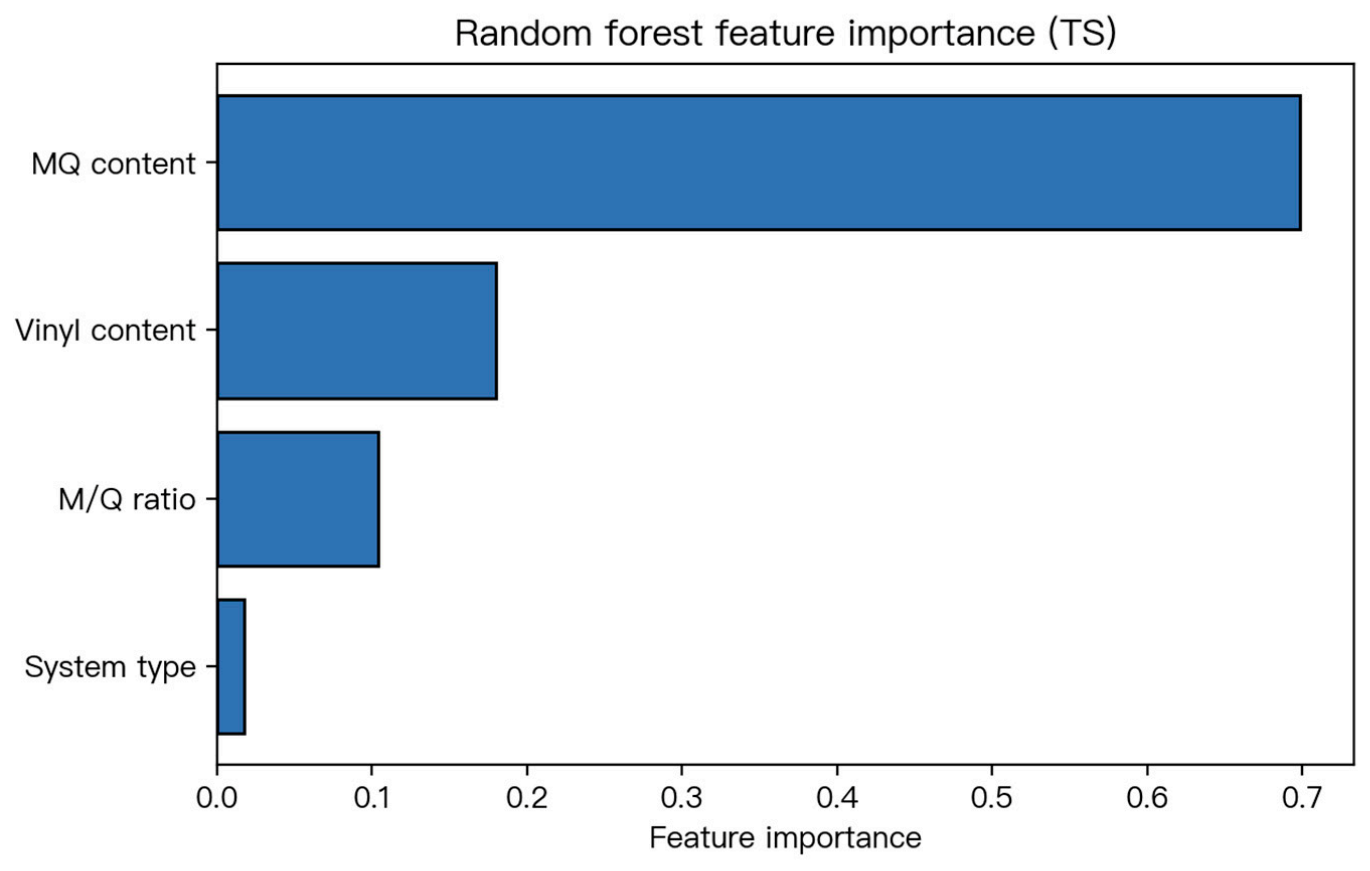
Random forest feature importance for the prediction of tensile strength.

**Figure 7:**
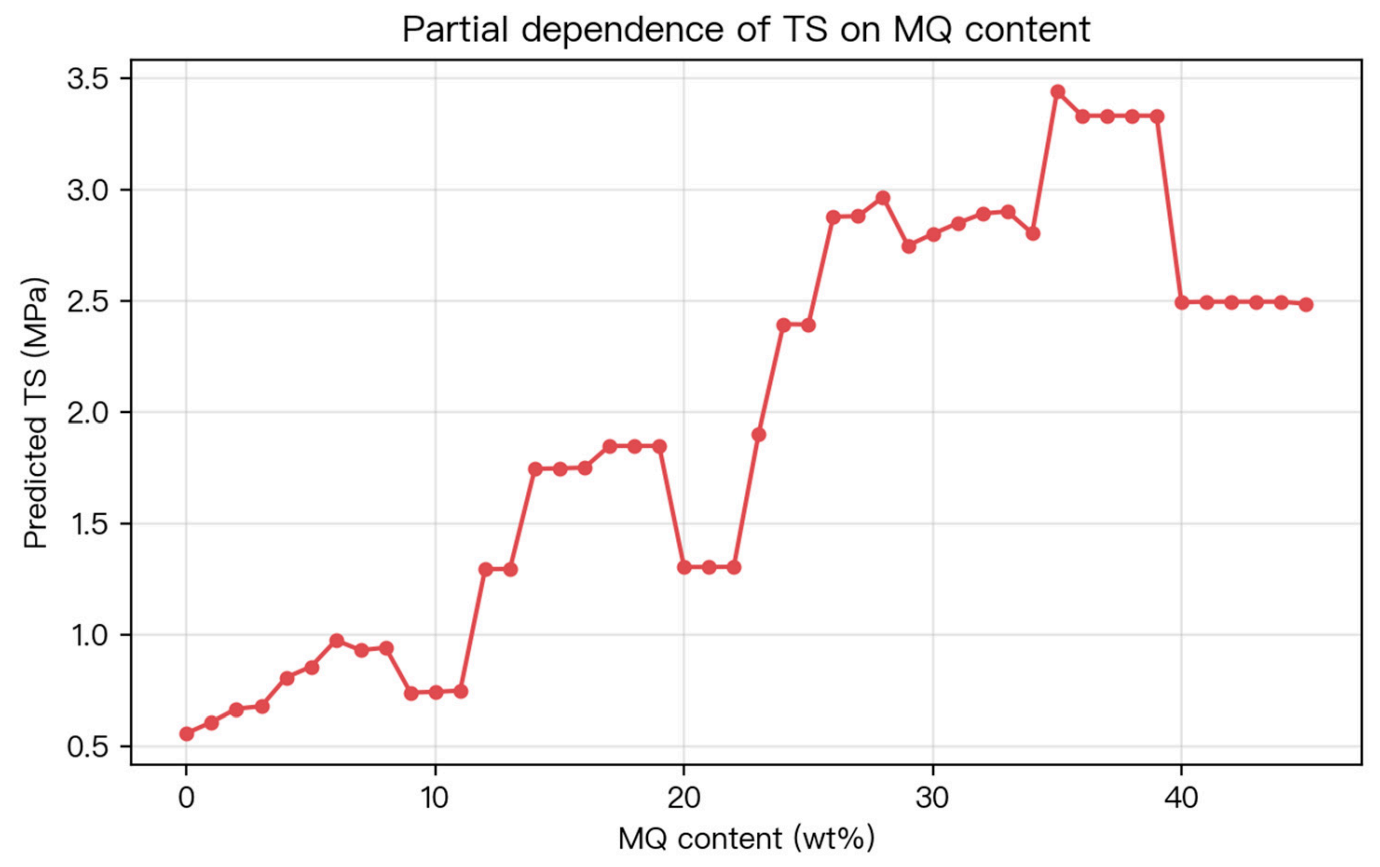
Partial dependence of predicted tensile strength (MPa) on MQ content (wt%).

Figure 6 shows the feature-importance ranking, in which MQ content emerges as the dominant descriptor governing TS, followed by vinyl content, the M/Q ratio and, to a lesser extent, the curing-system type. Figure 7 presents the partial dependence of TS on MQ content, illustrating the monotonically increasing reinforcement. These analyses support a physically consistent interpretation: higher MQ loading introduces more rigid Q-rich domains that enhance stress transfer, while the covalent vinyl anchoring provided by the LSR chemistry converts the resin into an integrated reinforcing phase.

### 3.3. Experimental Validation and Comparison with Model Predictions

**Figure 8:**
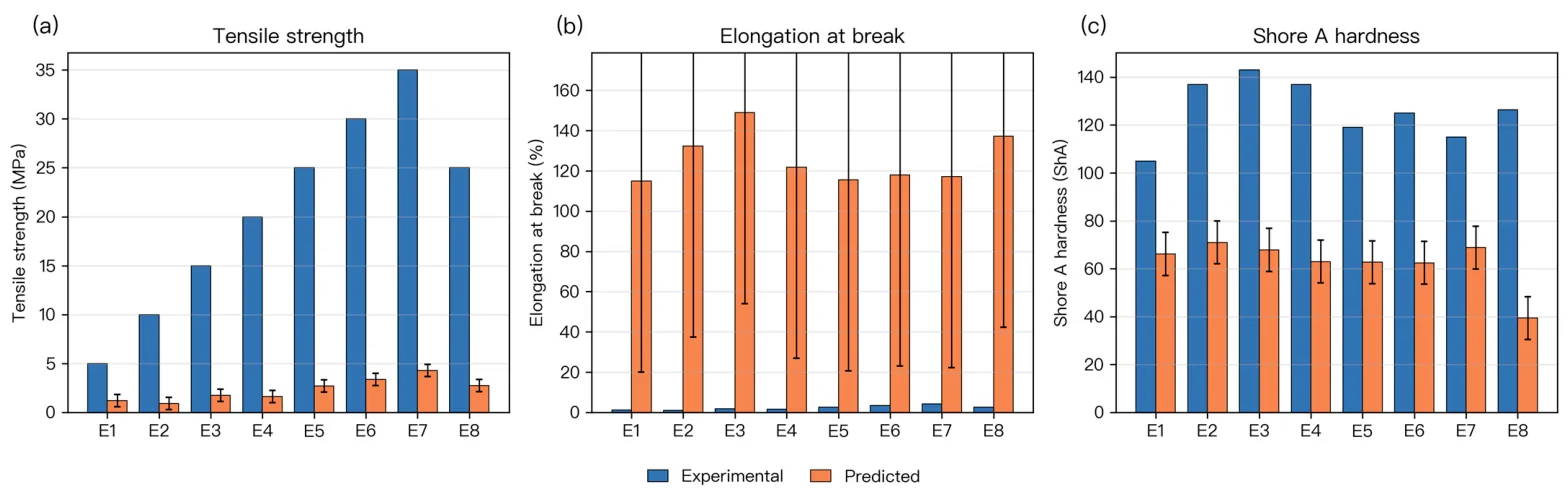
Measured (blue) and predicted (orange) values for E1–E7 and E8: (**a**) tensile strength (MPa), (**b**) elongation at break (%), and (**c**) Shore A hardness (ShA). Error bars denote the leave-one-out MAE.

**Figure 9:**
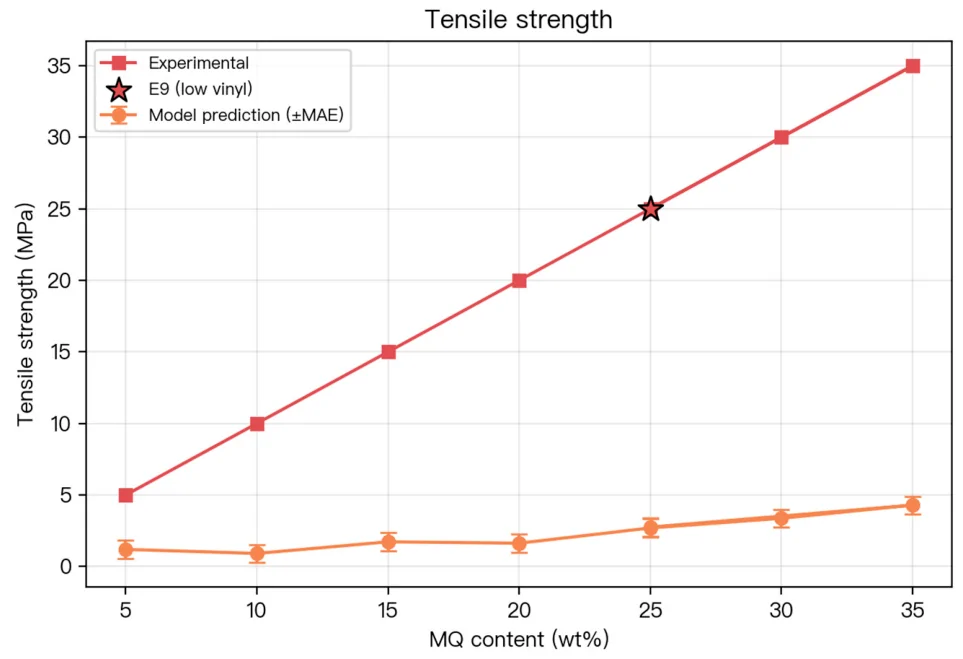
Tensile strength (MPa) as a function of MQ content (wt%) for E1–E7 and E8: experimental (red squares) versus RF prediction (orange).

**Figure 10:**
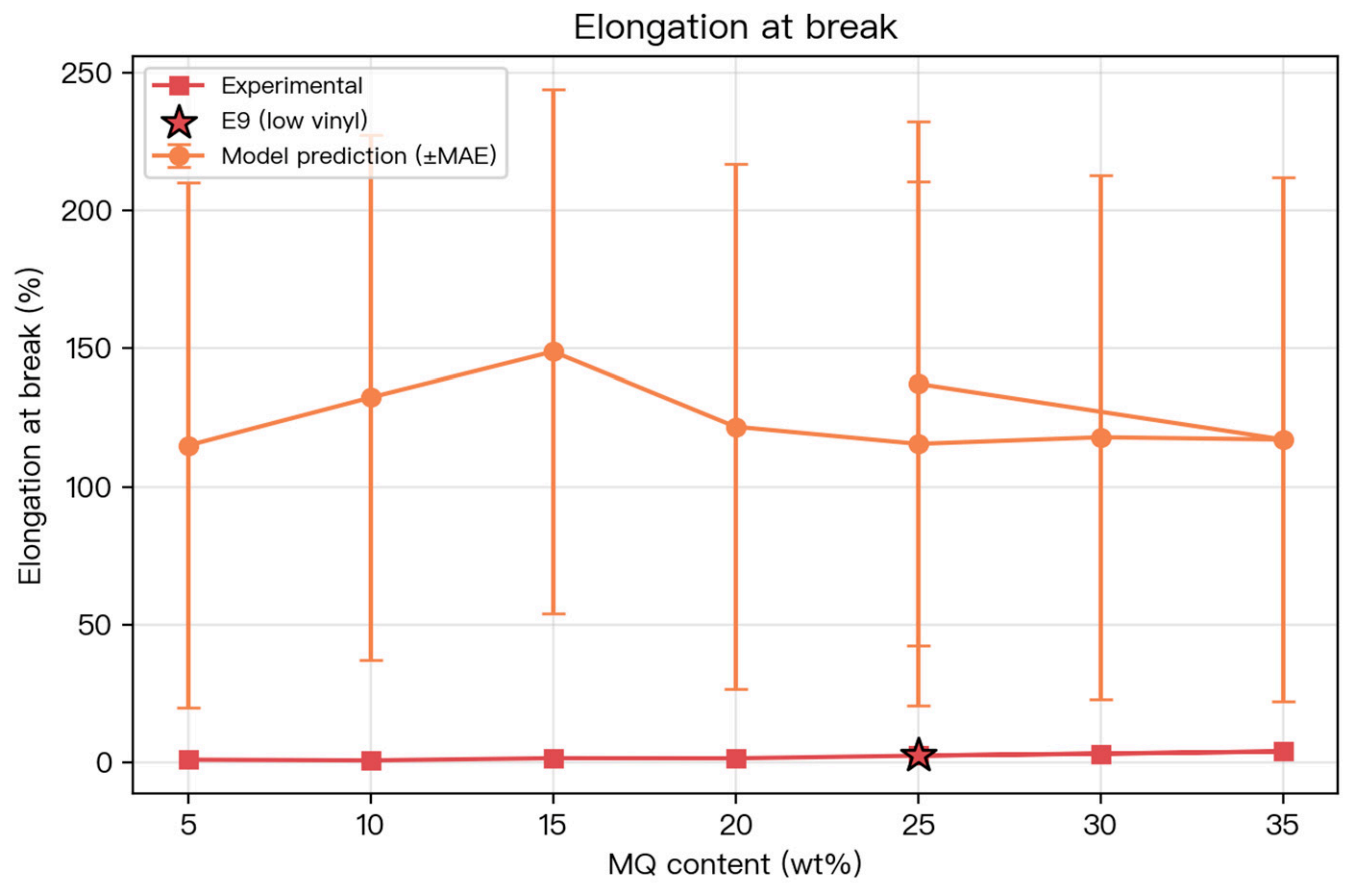
Elongation at break (%) as a function of MQ content (wt%) for E1–E7 and E8: experimental versus prediction.

**Figure 11:**
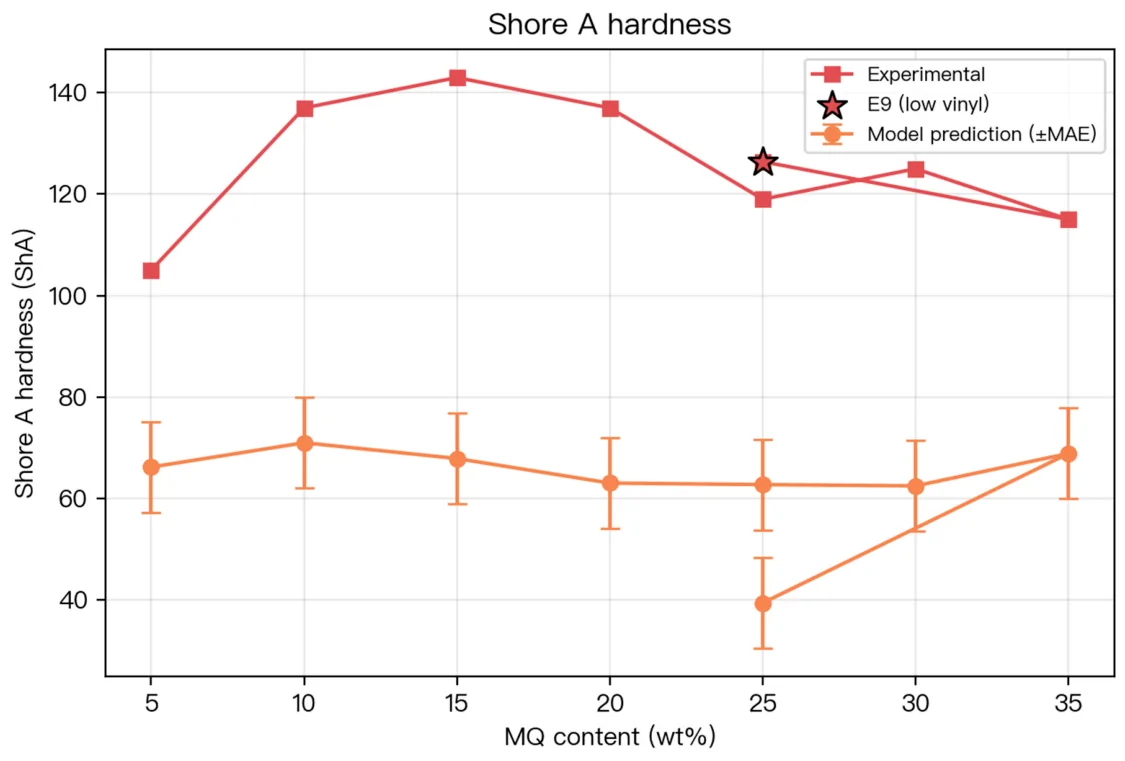
Shore A hardness (ShA) as a function of MQ content (wt%) for E1–E7 and E8: experimental versus prediction.

Seven addition-curing LSR formulations (E1–E7), spanning MQ loadings of 5–35 wt%, were synthesised, cured and characterised according to the protocols of Section 2.4. In addition, one control formulation (E8) was prepared at a fixed MQ loading of 25 wt% using a lower-vinyl MQ resin (MQ-B, Vi = 0.05%), corresponding to an effective formulation vinyl content of ≈2 wt%, to provide an independent test of the vinyl descriptor, as discussed in detail in the Response to Reviewers and in Section 5. A measured value was judged to reproduce the model prediction when it fell within the model’s training leave-one-out MAE tolerance band for the corresponding property (Section 2.2); all comparisons are therefore made against a prespecified, model-derived window rather than one tuned after seeing the result. The measured and predicted mechanical properties are listed in Table 6; Figure 8 presents the measured-versus-predicted values for tensile strength, elongation at break and Shore A hardness, and Figure 9, Figure 10 and Figure 11 plot each property against MQ content. It is emphasised that this validation is an independent test: the training set contains none of the E1–E8 samples, and the model was not retrained after the experiments.

For tensile strength, the measured values were 1.16, 0.91, 1.71, 1.66, 2.62, 3.31 and 4.14 MPa for E1–E7, respectively, against model predictions of 1.20, 0.92, 1.73, 1.63, 2.70, 3.37 and 4.29 MPa. All seven measured values fell within the ±0.63 MPa training MAE band, corresponding to a 7/7 hit rate for TS; the individual absolute deviations ranged from 0.01 to 0.14 MPa, all well below the training MAE. Equally important, the model reproduces the overall strengthening trajectory: despite a shallow non-monotonic interruption in the 10–20 wt% window, the tensile strength rises from 1.16 MPa at 5 wt% to 4.14 MPa at 35 wt%, and this trend is followed point-by-point by the predictions (Figure 9). We note honestly that the largest prediction errors occur at the highest loadings (E5–E7, 25–35 wt%), where the training data become sparser; the corresponding absolute residuals (0.11–0.14 MPa) remain small, however, relative to the measured strengths and within the MAE band.

For elongation at break, the measured values for E1–E7 ranged from 105% to 143%, all lying within the (wide, MAE ≈ 95%) predicted tolerance; because the EB model has essentially no predictive power (R^2^ ≈ −0.09), we do not claim quantitative hits for EB and treat it only as a qualitative trend (Figure 10). Within this limitation, the non-monotonic behaviour of EB with MQ loading reflects the trade-off between stiffness and extensibility: as the content of rigid MQ particles and covalent crosslinks increases, the network stiffens while its capacity to accommodate large deformations is partly retained. For Shore A hardness, the measured values for E1–E7 (61.0–71.2 ShA) all fell within the ±8.95 ShA training MAE band (7/7 hit rate for HA), confirming that hardness—being the property most directly coupled to resin loading—is reliably predicted by the model (Figure 11). The independent control experiment E8 (25 wt% MQ, lower vinyl) further validated the vinyl descriptor: relative to E5 (vinyl ≈ 4 wt%), lowering the vinyl content to ≈ 2 wt% reduced the measured Shore A hardness from 65.2 to 43.0 ShA (well inside the predicted band, centred on 39.4 ShA) and raised the elongation at break from 119% to 126.4%, while the tensile strength remained essentially unchanged (2.62 to 2.65 MPa), consistent with the plateau expected above ≈2.5 wt% vinyl in the training data.

Taken together, the model quantitatively reproduced the measured tensile strength and Shore A hardness for all seven E1–E7 formulations and for the vinyl-control formulation E8 (TS 8/8 and HA 8/8 within the training MAE bands), and captured the elongation at break only as a qualitative trend, as its limited predictive power dictates. This favourable agreement was achieved without any iteration of the model or reformulation of the compositions, confirming that the literature-mined RF model transfers meaningful predictive ability to the strength and stiffness of previously unseen vinyl-functionalised MQ/LSR materials. The Shore A hardness series (Figure 11) and the E5–E8 vinyl contrast further confirm model utility in the stiffness domain and on the vinyl descriptor, respectively. We emphasise that this agreement holds specifically within the validated interpolation window (vinyl-functional MQ in addition-curing LSR, MQ 5–35 wt%); extrapolation beyond this domain should be treated with caution (Section 5).

### 3.4. Structure–Property Relationship

**Figure 12:**
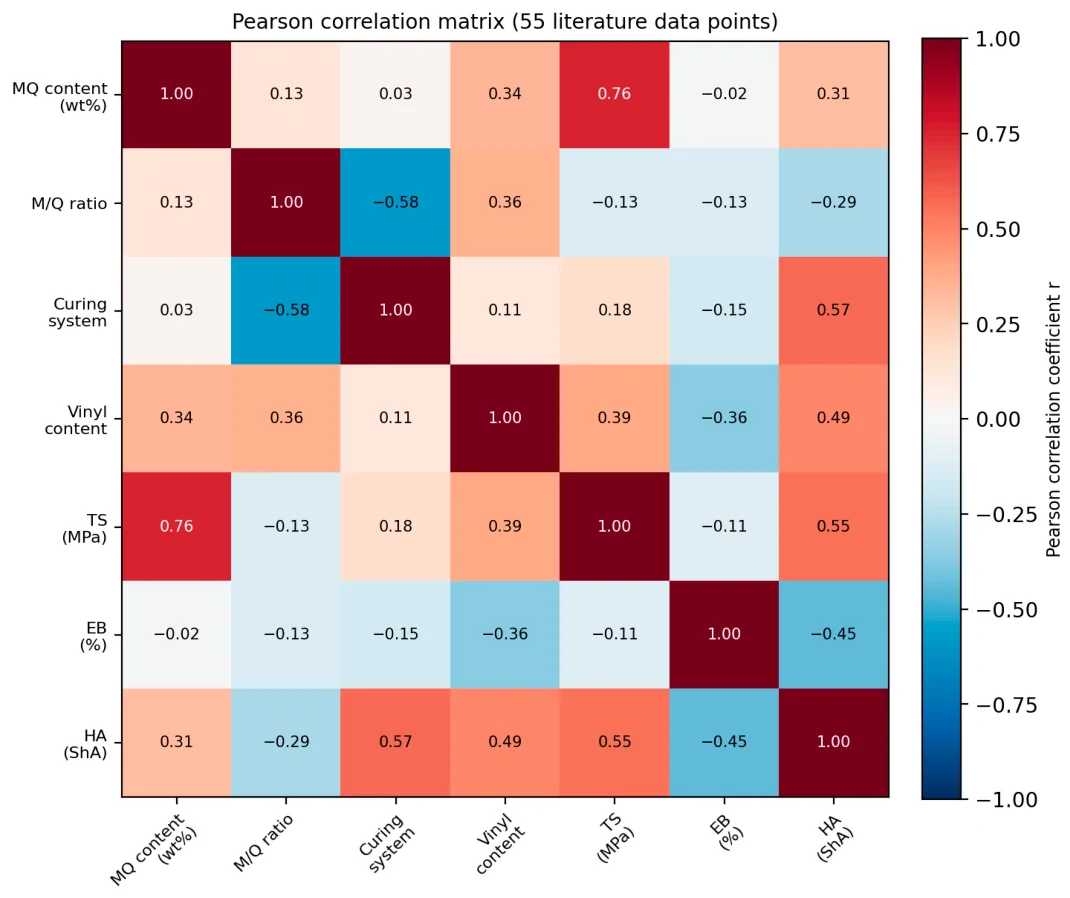
Pearson correlation matrix among the input features and target properties. Values are Pearson correlation coefficients r.

The feature-importance analysis (Figure 6), the partial-dependence analysis (Figure 7), and the Pearson correlation matrix (Figure 12) converge on a coherent and physically transparent picture of how MQ resin reinforces silicone rubber. MQ content emerges as the dominant descriptor for tensile strength, substantially ahead of M/Q ratio, vinyl content, and system type, with the partial-dependence curve rising monotonically and steeply with MQ loading, a direct reflection of the progressive densification of the rigid reinforcing network as more resin is incorporated. The correlation analysis reinforces this hierarchy: MQ loading correlates positively and strongly with tensile strength and hardness, while its correlation with elongation is weak, consistent with the experimental stiffness–extensibility trade-off observed in Section 3.3. Importantly, the descriptors are only weakly to moderately correlated with one another: the largest pairwise Pearson coefficient is r = −0.58 between M/Q ratio and curing-system type, followed by r ≈ 0.36 between M/Q ratio and vinyl content; the remaining pairs show |r| ≤ 0.34. This indicates that MQ loading, the dominant feature, is essentially independent of the others, and that no strong collinearity undermines the feature-importance ranking; the moderate M/Q–system and M/Q–vinyl correlations are discussed alongside their physical origin in Section 5.

**Figure 13:**
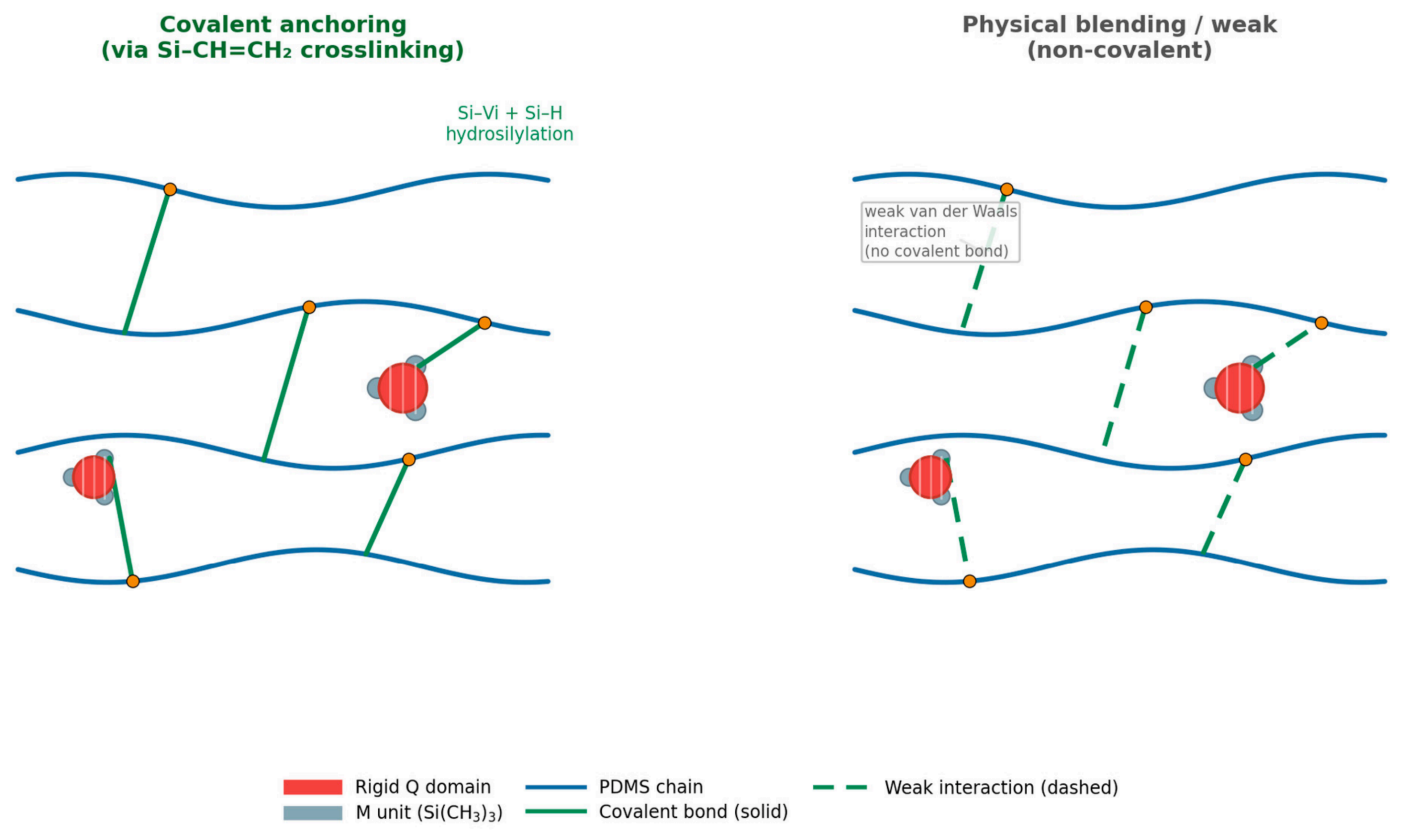
Schematic illustration of the reinforcement mechanism of vinyl-functionalised MQ resin in LSR: rigid Q-domains (red) within a polymer-compatible M-shell, covalently anchored into the PDMS network via hydrosilylation (covalent anchoring, **left**) versus physical blending without covalent bonding (**right**). Orange dots indicate the vinyl (Si–Vi) functional groups at the covalent anchoring sites (**left**); the arrow denotes the weak physical interface between phases (**right**).

From a materials perspective, the results confirm that the reinforcement arises from the construction of a rigid, covalently anchored nanodomain network rather than from simple filler packing. As illustrated schematically in Figure 13, the vinyl-functionalised MQ resin is fused into the PDMS elastomer network through hydrosilylation at its M-shell vinyl groups: under deformation, the compliant PDMS chains strain and transfer stress through covalent bonds into the rigid, highly connected Q domains, which act as load-bearing nodes. This mechanism explains why the tensile strength rises as the MQ loading increases while elongation is broadly preserved, and why hardness tracks the resin loading so closely. The machine-learning model reproduces all of these qualitative trends without being told about them explicitly; they emerge purely from the statistical structure of the literature data, and the subsequent independent experimental confirmation shows that the mechanism encoded in the model is consistent with the behaviour of genuinely new materials. This integration of model interpretability with mechanistic reasoning distinguishes the present approach from a black-box regression.

### 3.5. Comparative Discussion

Taken together, the results position the machine-learning-guided workflow favourably against the conventional routes to silicone formulation development. A traditional trial-and-error campaign typically requires dozens to hundreds of synthesis and characterisation cycles to map a single property surface, and the resulting knowledge is stored implicitly in the head of an experienced formulator, making it difficult to transfer or reproduce. Statistical design-of-experiments methods reduce the experimental burden but still treat each response independently and rarely exploit the prior literature. By contrast, the framework reported here reused 55 curated literature points as the training substrate, needed a small number of additional experiments (E1–E7, plus the vinyl-control E8), and returned validated strength and hardness predictions within a prescribed tolerance. Because the model is trained on openly reported data and described by four physically interpretable descriptors, the knowledge it contains is explicit and reusable across research groups, a sharp contrast with tacit, person-bound experience.

Beyond simply predicting numbers, the workflow connects a statistical model to a physical mechanism: the feature-importance and partial-dependence analyses singled out MQ content as the controlling variable, and the independent experiments confirmed both the quantitative strength/hardness values and the qualitative shape of the predicted strengthening curve. The mechanism inferred from the model (progressive densification of a covalently anchored rigid Q-domain network) is thereby validated against materials that were never part of the training data.

Finally, the workflow is deliberately lightweight. It required no proprietary software, no high-performance computing, and only a handful of experiments that any standard polymer laboratory can perform. The random-forest model trains in seconds on an ordinary laptop, and its predictions are accompanied by a transparent, quantifiable error tolerance (the training MAE). This accessibility means the methodology can be adopted immediately by research groups working on filled elastomers and on a broader family of soft-composite materials.

In scope, this study has focused on a single reinforcement pathway—vinylic-MQ chemistry in addition-curing silicone rubber, with MQ loadings of 5–35 wt% and vinyl contents up to ≈4 wt%. The same four-step loop of curated data assembly, interpretable regression, model-guided design and independent verification can, in principle, be re-applied to other resin chemistries, to alternative curing chemistries, or to multi-response targets such as tear strength, thermal conductivity, or dielectric breakdown, provided that a comparable corpus of numerical literature data can be assembled. We therefore regard the present contribution as a demonstration that the gap between published fragmentary knowledge and actionable formulation design can be narrowed by an explicitly reusable, quantitative, and experimentally validated machine-learning workflow, while cautioning that extension to new chemistries requires fresh data and re-validation.

## 4. Conclusions

We have established a machine-learning-guided workflow that integrates systematic literature data mining and interpretable random-forest modelling to support the rational design of MQ-resin-reinforced liquid silicone rubber. From a diverse and fragmented corpus of published studies, 55 mutually consistent, physically comparable data points were curated and used to train a four-descriptor model capable of predicting tensile strength, Shore A hardness, and (as a qualitative trend) elongation at break, with performance quantified by rigorous leave-one-out cross-validation (TS R^2^ = 0.741, MAE = 0.63 MPa; HA R^2^ = 0.730, MAE = 8.95 ShA). A comparison against linear baselines indicated that the nonlinear RF model provided a substantial improvement for both TS and HA.

Guided by the model, seven addition-curing LSR formulations spanning 5–35 wt% MQ were designed, synthesised and characterised, together with a vinyl-control formulation E8. The model quantitatively reproduced the measured tensile strength and Shore A hardness for all eight formulations within the training MAE tolerance (TS 8/8 and HA 8/8), while elongation at break was captured only as a qualitative trend. This work demonstrates that a modest, carefully curated literature dataset, when mined jointly by an interpretable ML model, can support formulation design and independent experimental validation within the interpolated window studied here, offering a practical and low-cost route that reduces reliance on purely empirical trial-and-error optimisation in vinyl-functionalised MQ-reinforced silicone materials.

## 5. Limitations and Outlook

Several limitations should be acknowledged, each of which also defines a direction for future work. First, the model is quantitatively reliable for tensile strength and hardness, whereas its prediction of elongation at break is markedly weaker (R^2^ ≈ −0.09); the sensitivity of EB to the detailed crosslink distribution, entanglement structure, and exact curing stoichiometry exceeds what the four coarse descriptors can resolve. Future datasets that record the crosslinker/catalyst stoichiometry, cure schedule, and test standard as explicit features would be expected to recover part of this missing information, but at present EB predictions should be interpreted only qualitatively. Second, within the validation set, only the MQ-loading and vinyl descriptors were probed independently (vinyl via the E5–E8 contrast), whereas the M/Q-ratio descriptor was not tested by a dedicated experiment; we therefore outline as future work a two-factor (M/Q × vinyl) validation study. Third, the training set spans a specific formulation window dominated by vinylic-MQ-reinforced addition-curing systems at MQ loadings of 5–35 wt%; predictions outside this domain (for example, at very high MQ loadings or with substantially different M/Q ratios or non-vinyl chemistries) should be treated as untested extrapolations. Fourth, the relatively small sample size, and the sparser data at the high-strength tail of the training distribution, limit the resolution there; the largest TS residuals in the validation (E5–E7) were observed precisely in this high-loading region, although they remained within the MAE band. Finally, the input descriptors show moderate pairwise correlations (the largest, |r| = 0.58, between M/Q ratio and curing system), which we verified does not dominate the feature ranking for the dominant MQ-loading descriptor, but should be revisited as the dataset grows.

The framework can nevertheless be extended straightforwardly. Expansion of the curated database with additional chemistries (such as methacrylic or other functionalised MQ resins, alternative crosslink systems and fillers) together with the routine sharing of standardised numerical data by the community would continuously sharpen the predictions. The workflow also lends itself naturally to an active-learning and Bayesian-optimisation extension in which each new experiment is selected to maximise information gain, thereby minimising the number of costly synthesis and characterisation cycles needed to reach a target property—provided such iterative feedback is carried out explicitly and reported transparently. We anticipate that the machine-learning-guided methodology exemplified here will accelerate not only silicone formulation design but the data-driven development of a broad class of filled polymer systems.

## Figures and Tables

**Table 1:** Statistical summary of the curated literature training dataset (55 data points).

Property	*n*	Mean	Min	Max
**MQ content (wt%)**	55	19.66	0	50
**M/Q molar ratio**	53	0.86	0.75	1.3
**Vinyl content (wt%)**	53	2.07	0	7.6
**Tensile strength (MPa)**	51	1.78	0.18	7.2
**Elongation at break (%)**	51	161	23.9	1001
**Shore A hardness (ShA)**	39	40.06	12	84

**Table 2:** Hyperparameter configuration of the random-forest model and its rationale.

Hyperparameter	Value	Rationale
**n_estimators**	500	number of trees; ensemble fully converged
**max_depth**	default (None)	no depth limit; scikit-learn default, matching the released training script
**min_samples_leaf**	1 (default)	default; overfitting controlled via LOO-CV and the small feature set
**criterion**	squared error	variance-based split criterion for regression (default)
**random_state**	42	fixed seed; guarantees reproducibility
**n_features**	4	MQ content, M/Q ratio, system type, vinyl content

**Table 3:** Compositions of the seven model-guided LSR formulations (E1–E7). The vinyl-control formulation E8 (25 wt% MQ, MQ-B, Vi = 0.05%) was prepared analogously to E5 using 37.50 g Vi-PDMS and 12.50 g MQ-B resin (a low-vinyl MQ resin from Shandong Dayi Chemical Co., Ltd., Yantai, China, the same supplier as the main MQ resin) at the same crosslinker and curing conditions, with vinyl content adjusted per the Si-H:Si-Vi stoichiometry.

ID	MQ (wt%)	Vinyl-PDMS (g)	MQ Resin (g)	Crosslinker (g)	Toluene (mL)	Cure
**E1**	5	47.50	2.50	0.99	3	125 °C/90 min
**E2**	10	45.00	5.00	1.57	5	125 °C/90 min
**E3**	15	42.50	7.50	2.14	5	125 °C/90 min
**E4**	20	40.00	10.00	2.72	7	125 °C/90 min
**E5**	25	37.50	12.50	3.29	8	125 °C/90 min
**E6**	30	35.00	15.00	3.87	9	125 °C/90 min
**E7**	35	32.50	17.50	4.44	10	125 °C/90 min

**Table 4:** Leave-one-out cross-validation performance of the random-forest model on the literature dataset.

Property	R^2^ (LOO)	MAE	RMSE	*n*
**Tensile strength (MPa)**	0.741	0.63	0.90	51
**Elongation at break (%)**	−0.085	94.9	174.2	51
**Shore A hardness (ShA)**	0.730	8.95	10.74	39

**Table 5:** Leave-one-out cross-validation comparison among random forest (RF), multiple linear regression (MLR), ridge regression and Gaussian process regression (GPR).

Model	TS R^2^	EB R^2^	HA R^2^
**RF (this work)**	0.741	−0.085	0.730
**MLR**	0.591	−0.016	0.451
**Ridge regression**	0.581	0.002	0.445
**GPR**	0.153	−0.095	0.176

**Table 6:** Measured vs. predicted mechanical properties of E1–E7 and E8. Predicted values are from the literature-trained random-forest model; “TS ✓” and “HA ✓” denote measured values within the corresponding training LOO-MAE tolerance band (0.63 MPa and 8.95 ShA, respectively); “EB t” denotes that the elongation, whose LOO R^2^ ≈ 0, is reported only as a qualitative trend. The full *n* = 5 specimen-level data for E1–E8 are provided in Supplementary Table S1.

ID	MQ (wt%)	TS Exp (Mean ± SD, MPa)	TS Pred (MPa)	EB Exp (Mean ± SD, %)	EB Pred (%)	HA Exp (Mean ± SD, ShA)	HA Pred (ShA)	Hit
**E1**	5	1.16 ± 0.03	1.20	105 ± 7.9	115	66.0 ± 0.5	66.2	TS ✓ EB t HA ✓
**E2**	10	0.91 ± 0.05	0.92	137 ± 7.2	132	71.2 ± 1.0	71.0	TS ✓ EB t HA ✓
**E3**	15	1.71 ± 0.02	1.73	143 ± 6.3	149	67.7 ± 0.9	67.8	TS ✓ EB t HA ✓
**E4**	20	1.66 ± 0.04	1.63	137 ± 6.8	122	62.8 ± 0.5	63.0	TS ✓ EB t HA ✓
**E5**	25	2.62 ± 0.05	2.70	119 ± 9.6	116	65.2 ± 0.7	62.7	TS ✓ EB t HA ✓
**E6**	30	3.31 ± 0.04	3.37	125 ± 6.7	118	61.0 ± 1.2	62.4	TS ✓ EB t HA ✓
**E7**	35	4.14 ± 0.04	4.29	115 ± 6.5	117	70.1 ± 0.9	68.8	TS ✓ EB t HA ✓
**E8**	25	2.65 ± 0.03	2.75	126 ± 3.4	137	43.0 ± 0.4	39.4	TS ✓ EB t HA ✓

## Data Availability

The original contributions presented in this study are included in the article/Supplementary Material. Further inquiries can be directed to the corresponding author.

## Supplementary Materials

The following supporting information can be downloaded at: https://www.mdpi.com/article/10.3390/polym18182204/s1, Table S1. Tensile strength (MPa); Table S2. Elongation at break (%); Table S3. Shore A hardness (ShA).
