# Supplementary material for: Machine Learning-Guided Design of MQ Silicone Resin Reinforced Addition-Curing Silicone Rubber: From Literature Data Mining to Experimental Validation
Source: Polymers (Basel). 2026 Sep 10;18(18):2204. doi: 10.3390/polym18182204 (PMC13610954; doi:10.3390/polym18182204)
Supplement: Supplementary file 1 [file polymers-18-02204-s001.zip › polymers-4539490-supplementary.pdf]

Supplementary Tables S1–S3. Individual specimen measurements (n = 5) for the eight validation formulations E1–E8.

All formulations were addition-cured at 125 °C for 90 min with a fixed solid content (toluene 3–10 mL per 50 g). TS, tensile strength; EB, elongation at break; HA, Shore A hardness. Each value is a single specimen reading; the mean ± standard deviation (n = 5) is reported in Table 6 of the main text.

Supplementary Table S1. Tensile strength (MPa)

ID	TS1	TS2	TS3	TS4	TS5	Mean ± SD
**E1**	1.160	1.140	1.190	1.120	1.170	**1.156±0.027**
**E2**	0.860	0.910	0.950	0.880	0.970	**0.914±0.046**
**E3**	1.700	1.740	1.680	1.720	1.690	**1.706±0.024**
**E4**	1.630	1.680	1.710	1.600	1.660	**1.656±0.043**
**E5**	2.560	2.610	2.640	2.680	2.590	**2.616±0.046**
**E6**	3.280	3.340	3.310	3.260	3.370	**3.312±0.044**
**E7**	4.110	4.160	4.090	4.140	4.190	**4.138±0.040**
**E8**	2.630	2.660	2.610	2.690	2.640	**2.646±0.030**

Supplementary Table S2. Elongation at break (%)

ID	EB1	EB2	EB3	EB4	EB5	Mean ± SD
**E1**	98	104	111	97	115	**105.0±7.9**
**E2**	132	141	147	129	138	**137.4±7.2**
**E3**	139	147	152	136	143	**143.4±6.3**
**E4**	132	141	129	146	137	**137.0±6.8**
**E5**	106	118	127	113	129	**118.6±9.6**
**E6**	116	124	130	122	133	**125.0±6.7**
**E7**	107	115	120	111	123	**115.2±6.5**
**E8**	124	129	122	127	130	**126.4±3.4**

Supplementary Table S3. Shore A hardness (ShA)

ID	HA1	HA2	HA3	HA4	HA5	Mean ± SD
**E1**	65.4	66.2	65.8	66.7	66.1	**66.0±0.5**
**E2**	70.6	71.9	72.4	69.8	71.5	**71.2±1.0**
**E3**	66.6	67.1	67.8	68.4	68.7	**67.7±0.9**
**E4**	62.4	63.2	62.8	63.5	62.2	**62.8±0.5**
**E5**	64.6	65.1	65.8	64.4	66.0	**65.2±0.7**
**E6**	59.2	60.5	61.1	61.9	62.3	**61.0±1.2**
**E7**	68.8	69.6	70.3	70.9	71.0	**70.1±0.9**
**E8**	42.7	43.2	42.5	43.6	42.9	**43.0±0.4**
